# Correction to “Low-Temperature Structural Battery Electrolytes Produced by Polymerization-Induced Phase Separation”

**DOI:** 10.1021/acsapm.4c02519

**Published:** 2024-09-04

**Authors:** Sayyam Deshpande, Vishaal Vidyaprakash, Suyash Oka, Smita S. Dasari, Kai-Wei Liu, Chen Wang, Jodie L. Lutkenhaus, Micah J. Green

In our original article, experiments were carried out according to ASTM D638 to find Young’s modulus of structural battery electrolytes using tensile testing. Type V dogbones were used during testing. We did not recognize that the wrong gauge length was used during the calculations until it was pointed out by one of our students after the publication of our paper. The corrected modulus values decreased by a factor of 2.86 while the energy to break increased by a factor of 2.86.

Due to this calculation mistake, Figures 6, 8, and 9 are incorrect in the original article. The correct figures are here.

**Figure 6:**
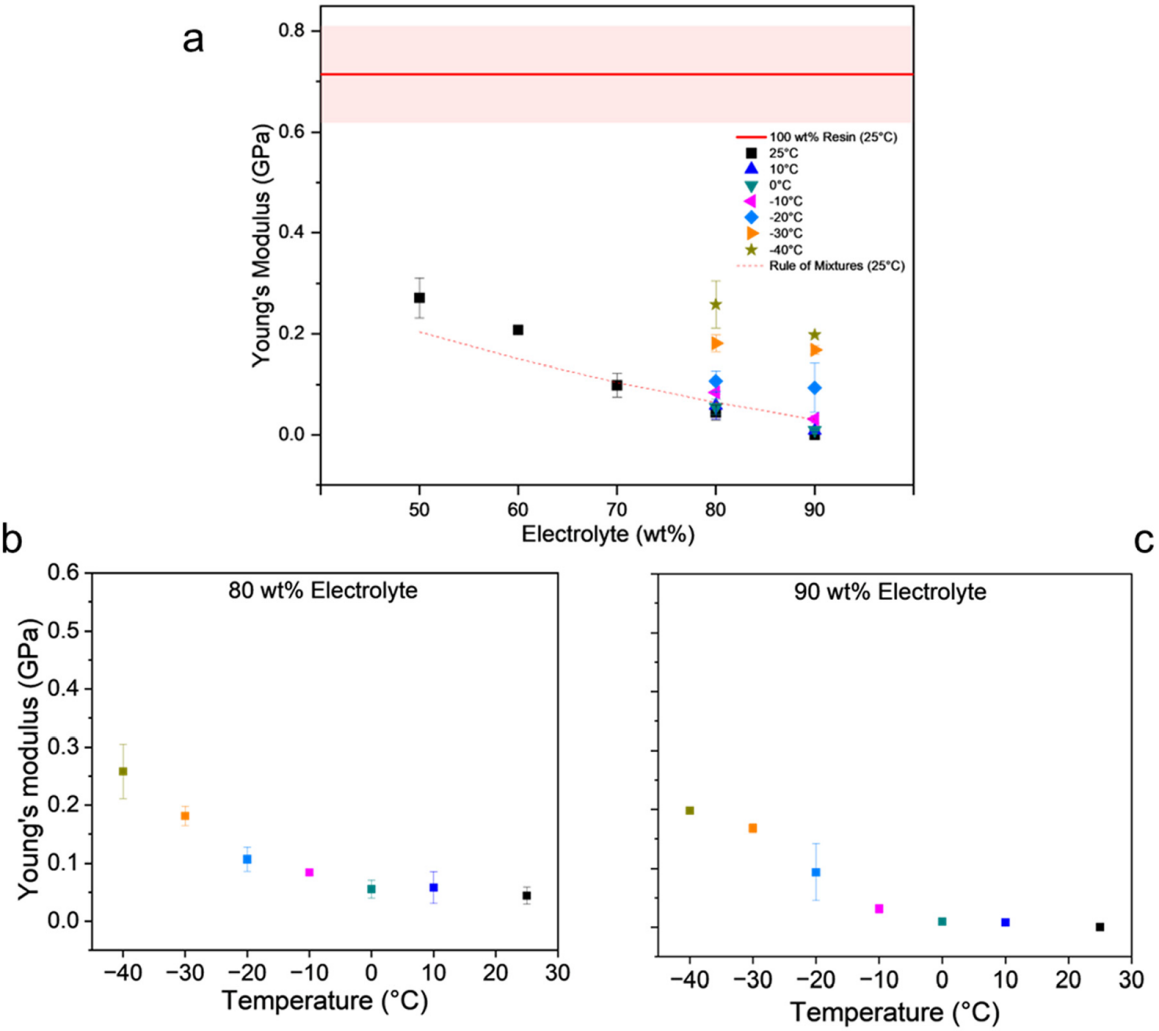
Young’s modulus (a) as a function of electrolyte concentration at different temperatures, (b) as a function of temperature for 80% electrolyte concentration, and (c) as a function of temperature for 90% electrolyte concentration. The red stripe in (a) represents the pure resin modulus at room temperature (where the center line is the mean value, and the shaded region is the associated error).

**Figure 8:**
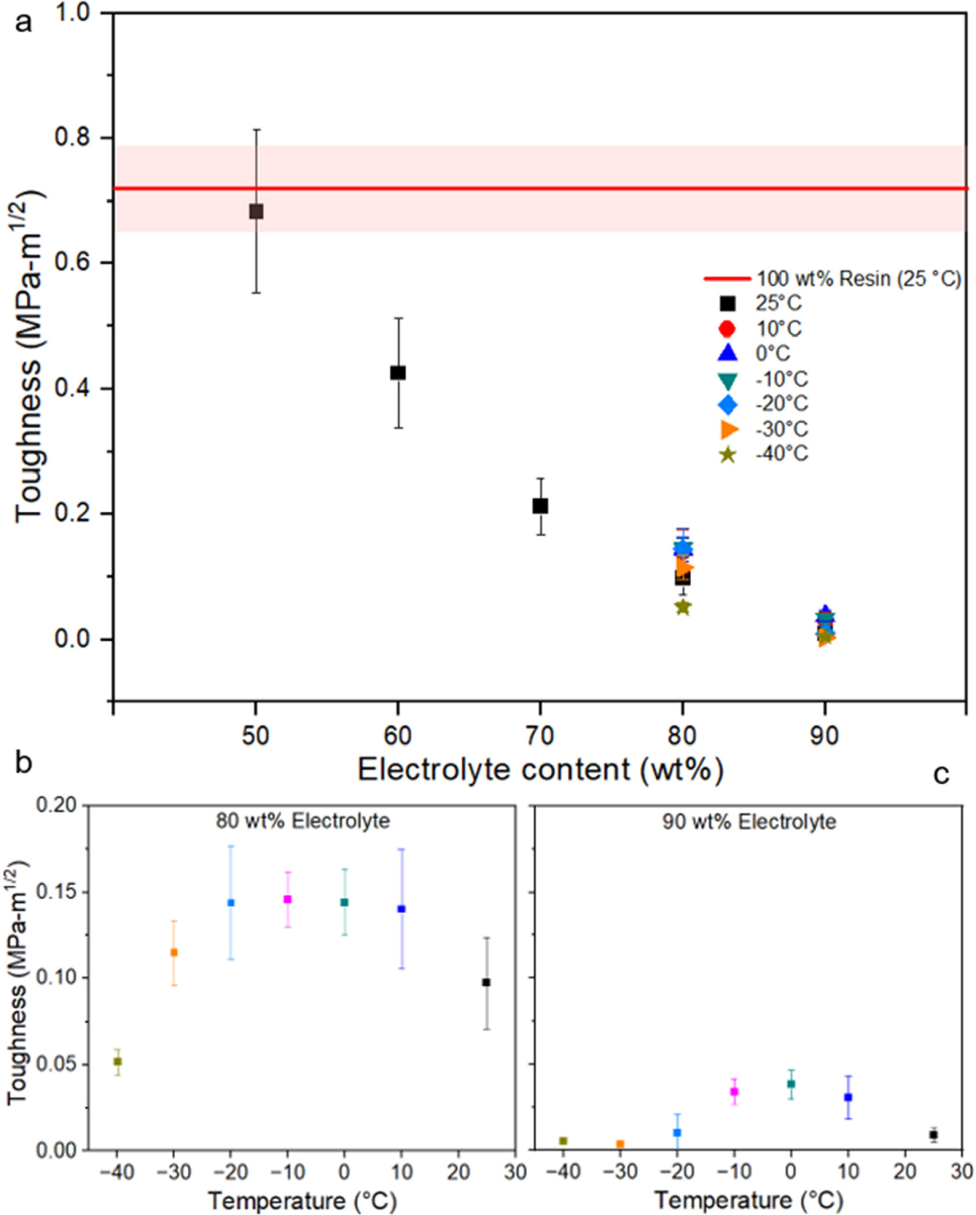
Toughness (a) as a function of electrolyte concentration at different temperatures, (b) as a function of temperature for 80% electrolyte concentration, and (c) as a function of temperature for 90% electrolyte concentration. The red stripe in (a) represents the pure resin’s ultimate tensile strength at room temperature (where the center line is the mean value and the shaded region is the associated error).

**Figure 9:**
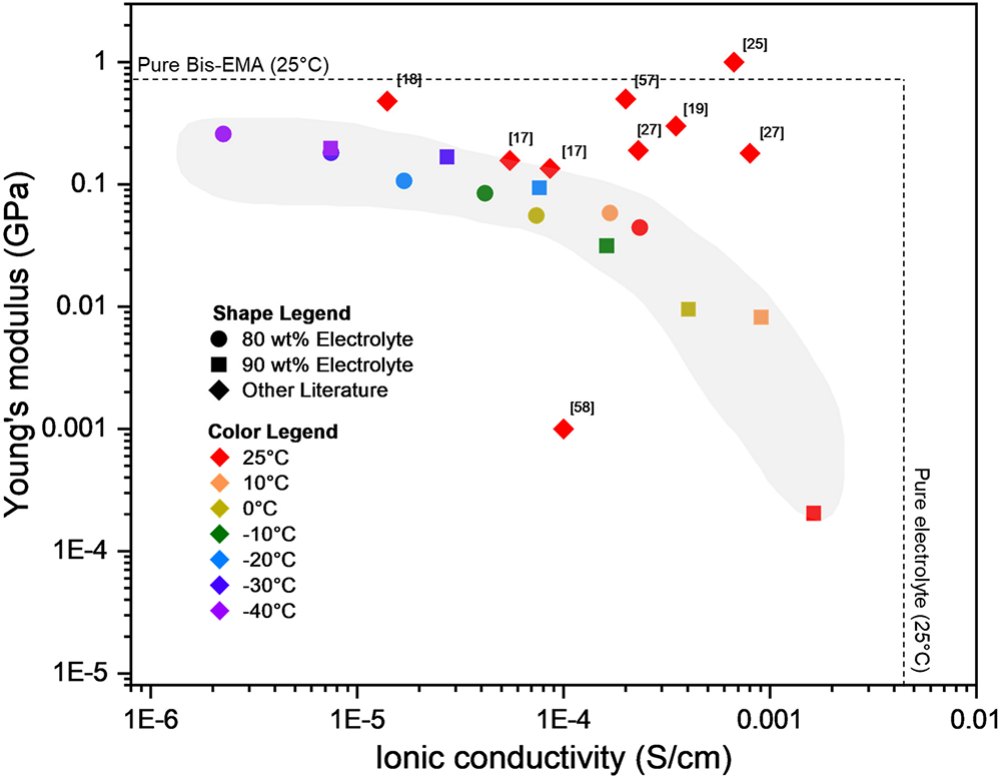
Multifunctionality graph: modulus vs ionic conductivity for SBEs. The dashed lines indicate the properties of the pure resin and electrolyte. Our data for 80 and 90 wt % electrolyte are shown as circles and squares, respectively, in the gray shaded region; the data show the trade-off between the two properties. Diamonds indicate representative bicontinuous electrolytes for batteries and supercapacitors from the literature.

Also, in the original Supporting Information, Figures S15, S16, S17, and S18 are incorrect. The correct figures are in the revised Supporting Information here.

Some text in the original article also needs corrected. In the Abstract, the phrase “...the modulus decreased from 0.910 GPa to 8.13 × 10^–4^ GPa at 25 °C...” should be changed to “...the modulus decreased from 0.272 GPa to 2.05 × 10^–4^ GPa at 25 °C...”.

On page 6327, the sentence “As expected, the moduli increase steadily from 8.13 × 10^–4^ GPa at 25 °C to 0.359 GPa at −40 °C for the 90 wt % electrolyte sample and 0.112 GPa at 25 °C to 0.444 GPa at −40 °C for the 80 wt % electrolyte sample.” should be changed to “As expected, the moduli increase steadily from 2.05 × 10^–4^ GPa at 25 °C to 0.198 GPa at −40 °C for the 90 wt % electrolyte sample and 0.044 GPa at 25 °C to 0.258 GPa at −40 °C for the 80 wt % electrolyte sample.”

On page 6329, the phrase “At 25 °C, the 80 wt % sample has a modulus of 0.11 GPa...” should be changed to “At 25 °C, the 80 wt % sample has a modulus of 0.044 GPa...”, and the phrase “Similarly, the 90 wt % sample at −10 °C with a modulus of 0.07 GPa...” should be changed to “Similarly, the 90 wt % sample at −10 °C with a modulus of 0.032 GPa...”.

In the Conclusions, the phrase “...modulus of 0.07 GPa at a temperature of −10 °C.” should be changed to “...modulus of 0.032 GPa at a temperature of −10 °C.”, and the phrase “...a modulus of 0.11 GPa.” should be changed to “...a modulus of 0.044 GPa.”

These changes do affect the values of modulus and energy to break; however, they do not change the trend of the data. Therefore, the conclusions of the original article are not changed.

The authors apologize for any inconvenience these changes have caused.

## Supporting Information Available

Digital images of curing in the glovebox, coin cell assembly schematic, FT-IR on SBEs, digital images of dogbone samples, porosity, and tortuosity values, bode plots, Nyquist plots, EIS circuit model, Arrhenius equation, fit and fit parameters, raw ionic conductivity data, DSC data, raw tensile stress–strain curves, density data, specific modulus data, literature comparison of Young’s modulus and ionic conductivity data, and GCD cycles for low-temperature battery tests (PDF)

