# Supplementary material for: Correction to “Low-Temperature Structural Battery Electrolytes Produced by Polymerization-Induced Phase Separation”
Source: ACS Appl Polym Mater. 2024 Sep 4;6(17):11066–9. doi: 10.1021/acsapm.4c02519 (PMC11406479; doi:10.1021/acsapm.4c02519)
Supplement: Supplementary file 1 — ap4c02519_si_001.pdf [file ap4c02519_si_001.pdf]

<u>**SUPPORTING INFORMATION**</u>

Low-temperature structural battery electrolytes produced by polymerization-induced phase separation

Sayyam Deshpande[a,1], Vishaal Vidyaprakash[a,1], Suyash Oka[a], Smita S. Dasari[a], Kai-Wei Liu[c], Chen Wang[a], Jodie L. Lutkenhaus,[a,b*] and Micah J. Green[a,b*]

Affiliations:

[a]*Artie McFerrin Department of Chemical Engineering, Texas A&M University, College Station, Texas 77843, USA*

[b]*Department of Material Science and Engineering, Texas A&M University, College Station, Texas 77843, USA*

[c]*Texas A&M Transportation Institute, Texas A&M University, College Station, Texas 77843, USA*

[1]*Indicates equal author contribution*

***Corresponding author's email: micah.green@tamu.edu, jodie.lutkenhaus@tamu.edu**

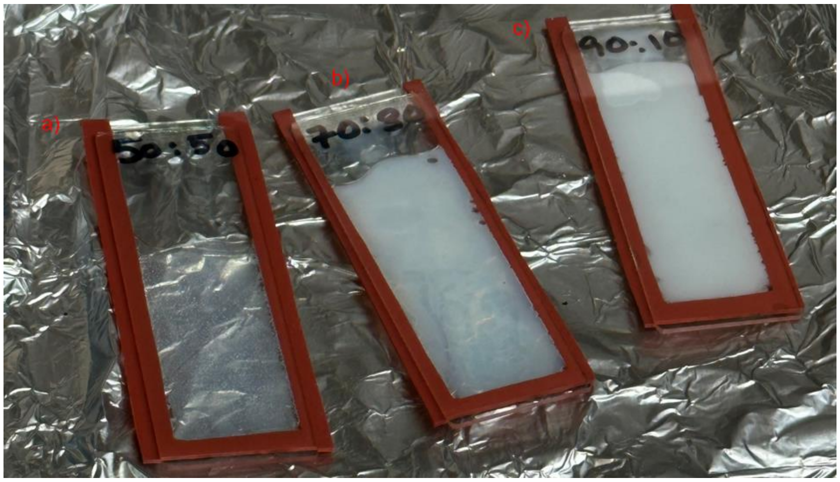

Figure S1: Curing of samples at 80 ℃ in a glovebox on a hot plate. The corresponding compositions are a) 50 wt% electrolyte, b) 70 wt% electrolyte, and c) 90 wt% electrolyte. The dimensions of the molds are 7.5 cm x 2.6 cm x 0.1 cm.

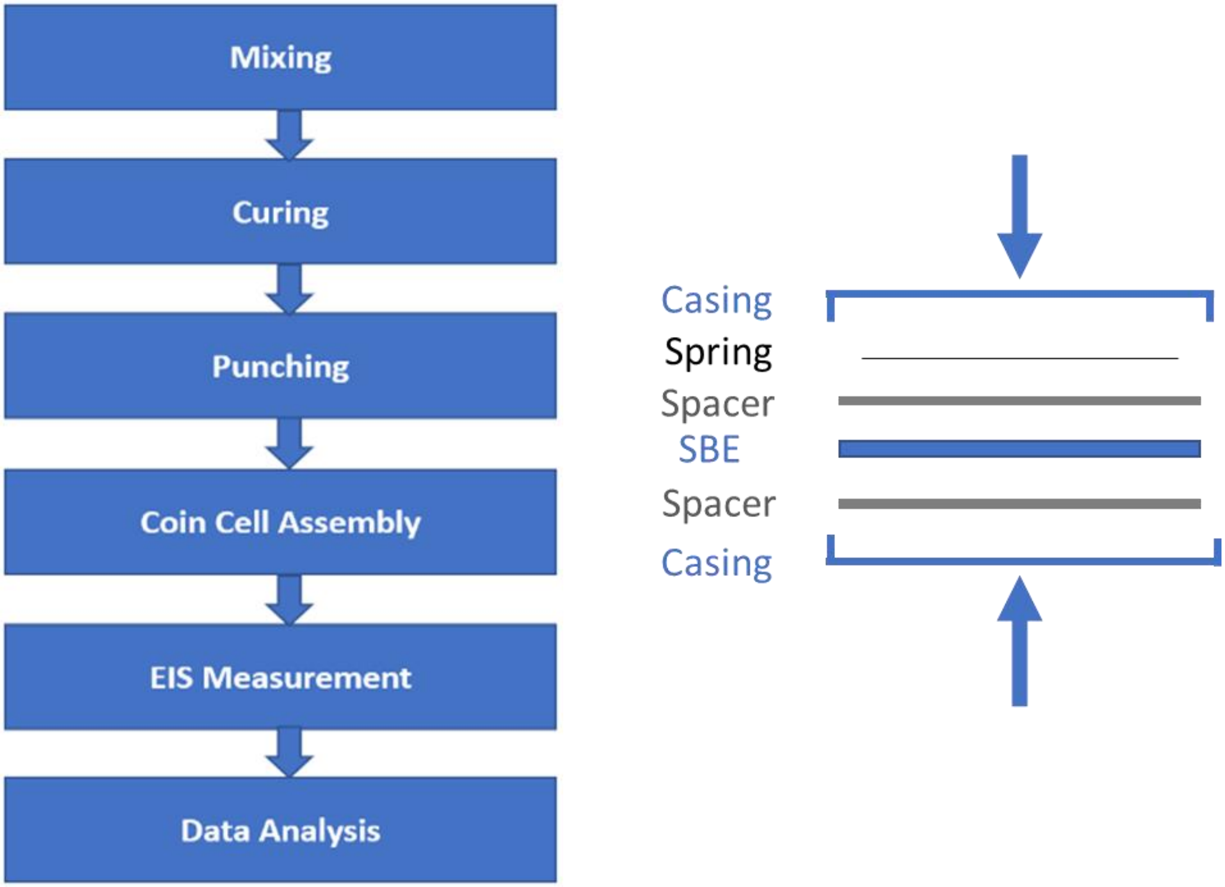

Figure S2. Structural battery electrolyte (SBE) fabrication and characterization process (left) and coin cell assembly (right).

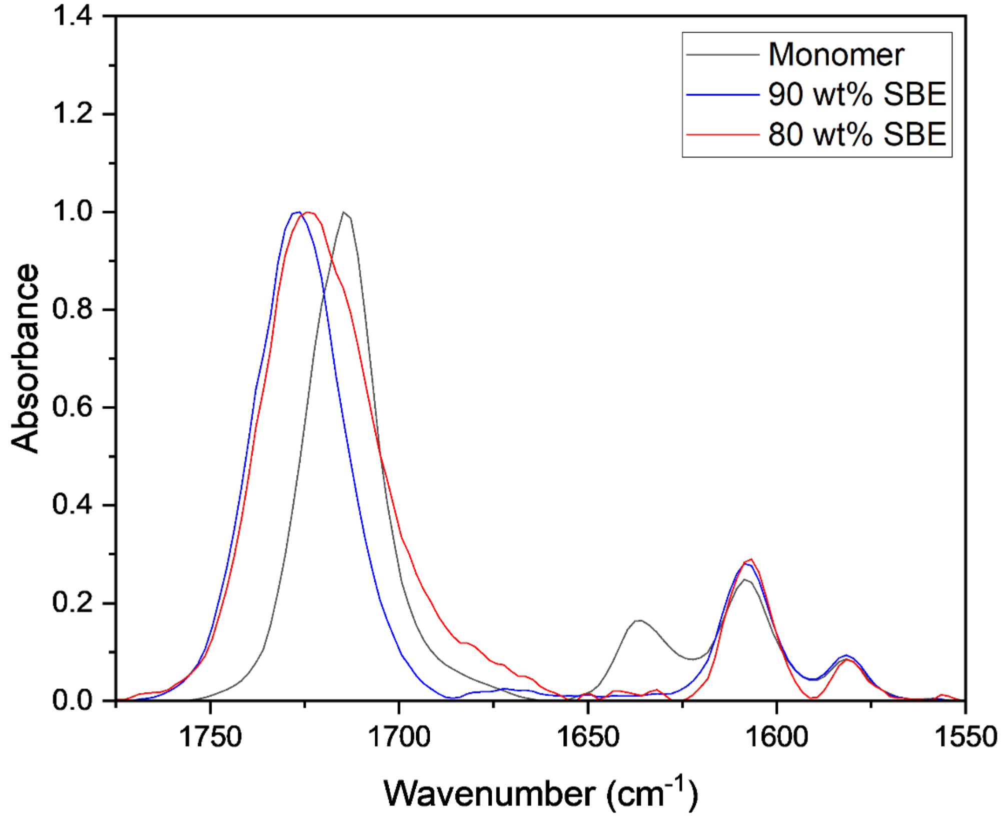

Figure S3. FT-IR spectra of monomer, 80 wt% SBE and 90 wt% SBE used to evaluate degree of cure. All samples were prepared outside the glovebox.

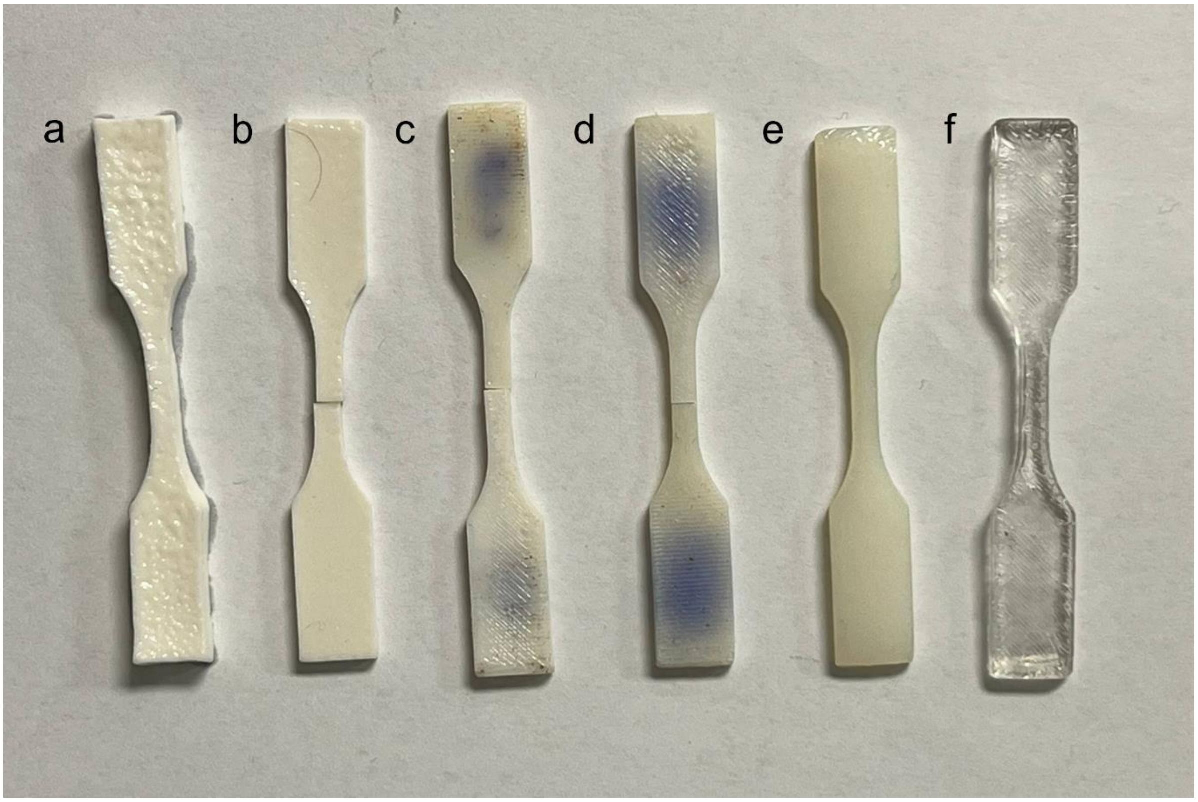

Figure S4. Digital images of structural battery electrolyte (SBE) dogbone samples corresponding to compositions of a) 90 wt% electrolyte, b) 80 wt% electrolyte, c) 70 wt% electrolyte, d) 60 wt% electrolyte, e) 50 wt% electrolyte, and f) pure resin. A change in opacity is observed with decreasing electrolyte content. The width of each sample is 3 mm. Note: The blue color is due to the ink used to mark the samples.

Table S1: Effective porosities of SBE samples calculated using **Equation 2**. Tortuosity is calculated using **Equation 3**.

Composition (wt% electrolyte)	Porosity (ε)	Tortuosity (τ)
90 wt%	0.823	1.80
80 wt%	0.569	8.62
70 wt%	0.413	17.5
50 wt%	0.372	31.9

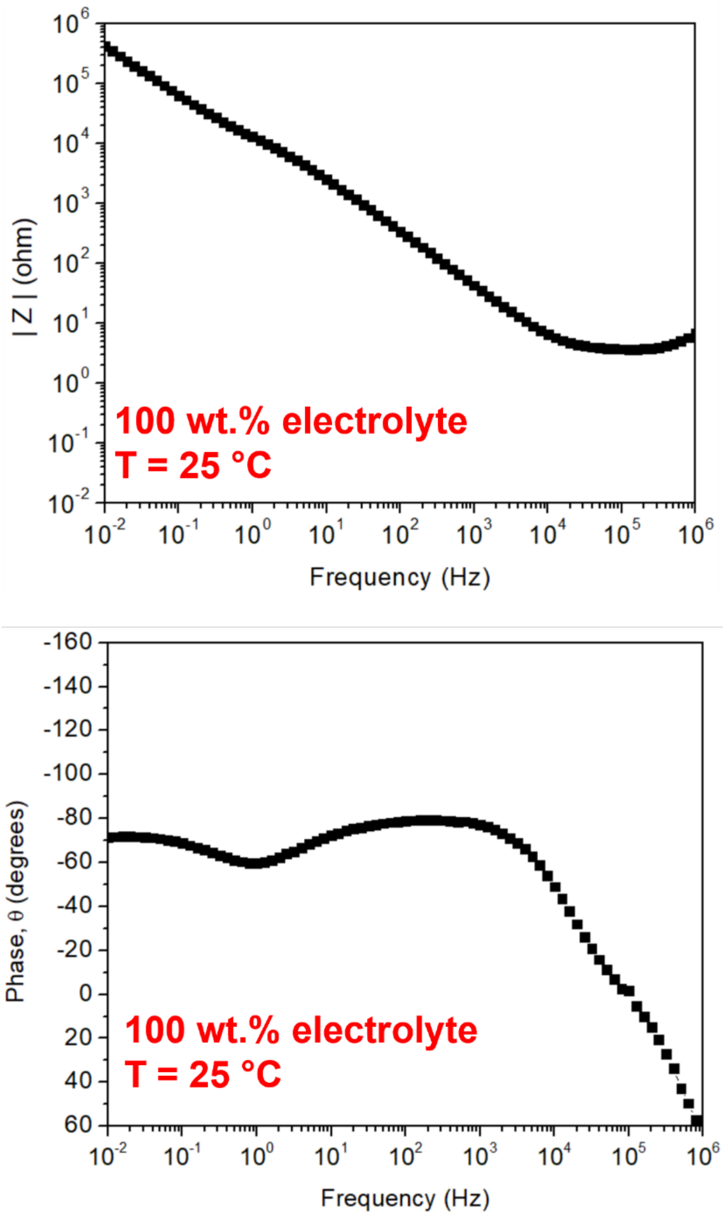

Figure S5: Bode plot of samples at 25 ℃ corresponding to composition of 100 wt% electrolyte.

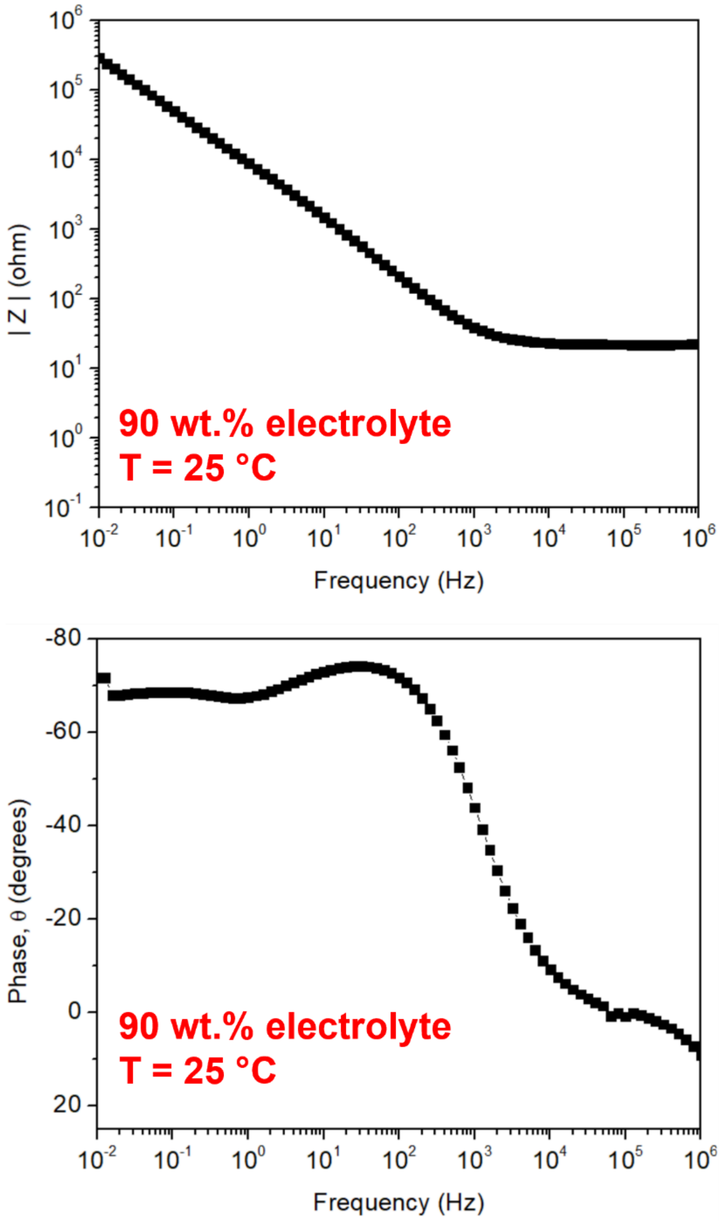

Figure S6: Bode plot of structural battery electrolyte (SBE) at 25 ℃ corresponding to composition of 90 wt% electrolyte.

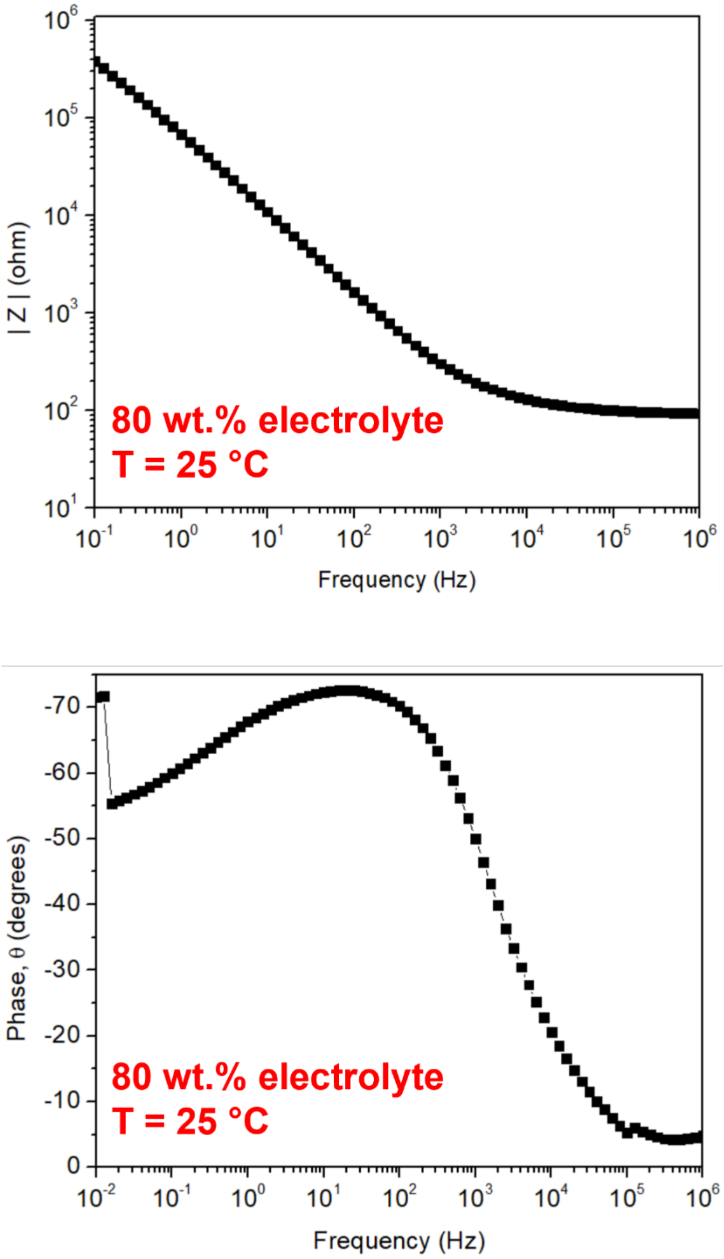

Figure S7: Bode plot of structural battery electrolyte (SBE) at 25 °C corresponding to composition of 80 wt% electrolyte.

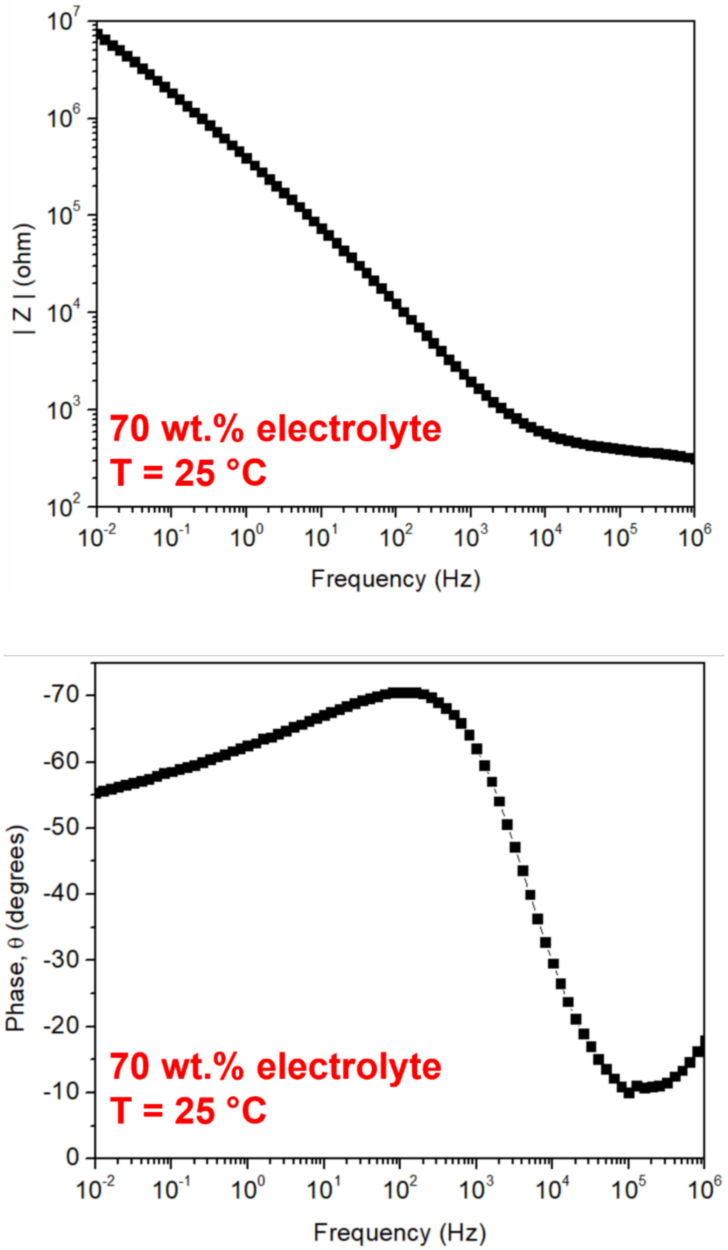

Figure S8: Bode plot of structural battery electrolyte (SBE) at 25 °C corresponding to composition of 70 wt% electrolyte.

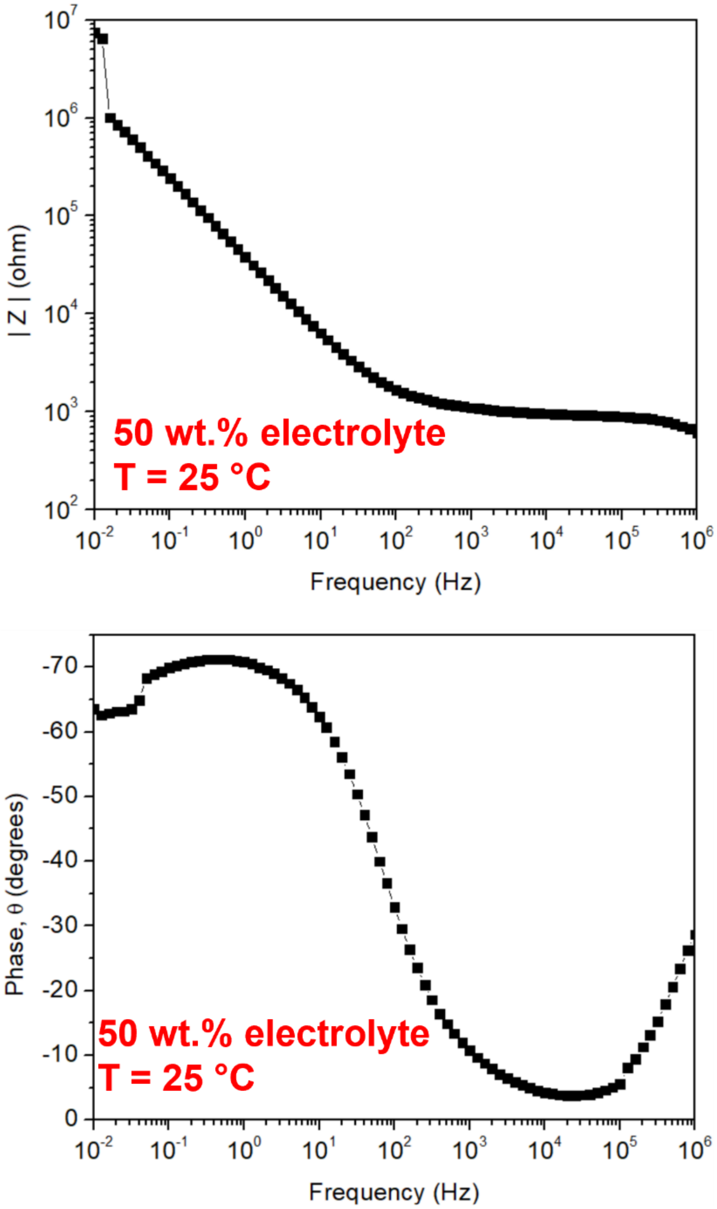

Figure S9: Bode plot of structural battery electrolyte (SBE) at 25 °C corresponding to composition of 50 wt% electrolyte.

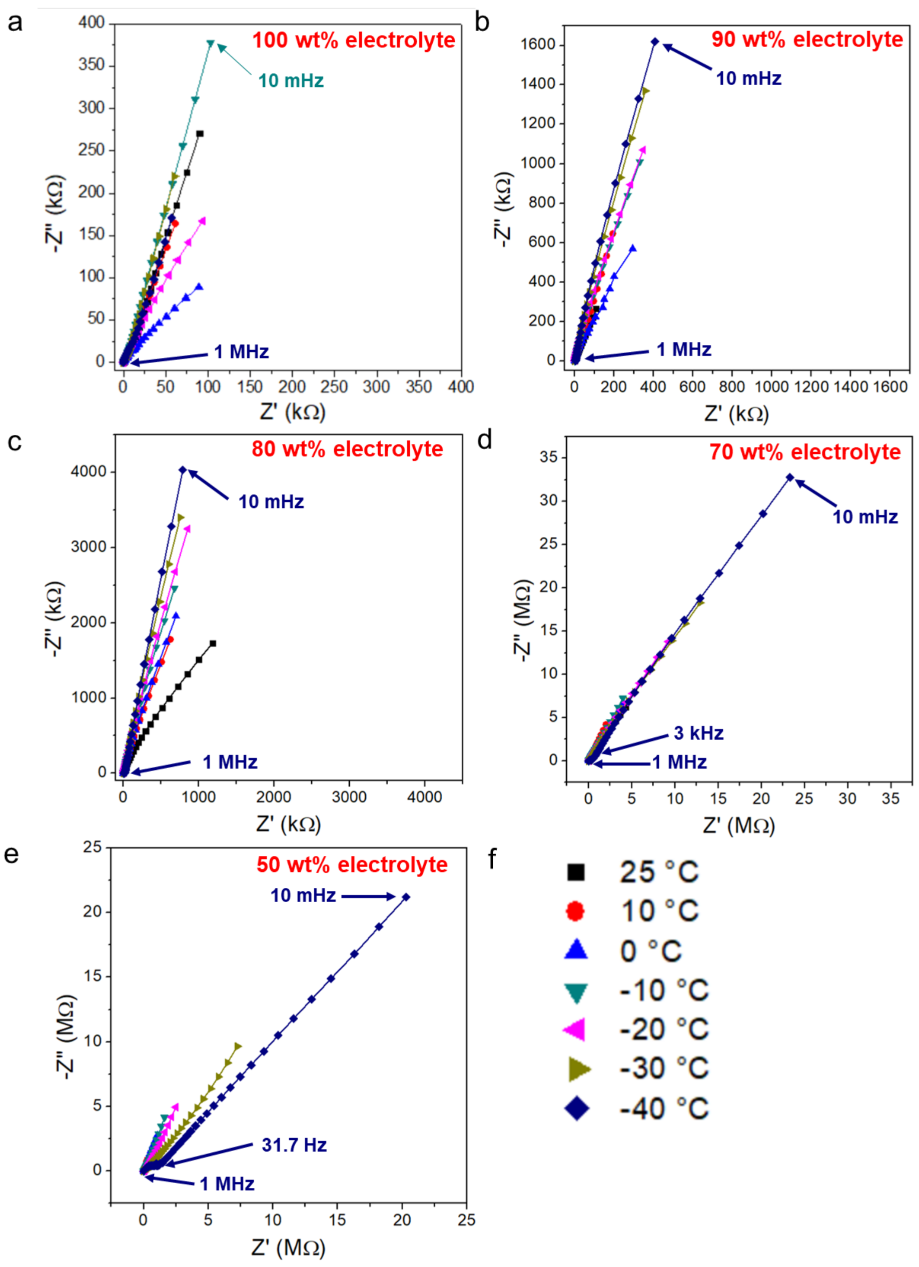

Figure S10: Nyquist plots showing the variation in impedance for different samples with temperature: a) 100 wt% electrolyte, b) 90 wt% electrolyte, c) 80 wt% electrolyte, d) 70 wt% electrolyte, e) 50 wt% electrolyte, and f) legend.

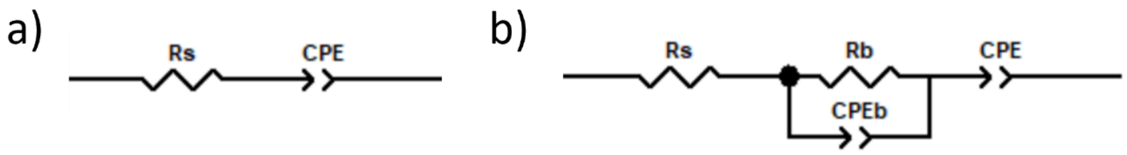

Figure S11: Equivalent circuit models used to fit the EIS data. The circuits corresponding to (a) represent the SBEs without any distinct bulk resistance and (b) SBEs with distinct bulk resistance. Circuit (a) represents a resistor in series with a constant phase element in which the resistor Rs represents the equivalent series resistance of the electrolyte/SBE and spacer and the constant phase element CPE represents the non-ideal capacitive component of impedance of the spacer. Circuit (b) represents a resistor in series with a constant phase element and a parallel combination of a resistor and a constant phase element. The resistor R_s represents the resistance of the stainless-steel spacer, resistor R_b represents the resistance of the SBE, constant phase element CPE_b represents non-ideal capacitance of the SBE, and CPE represents the non-ideal capacitive component of impedance of the spacer. Circuit (a) was used to fit the data for the 100 wt% electrolyte sample.

Section S1:

The Arrhenius dependence of ionic conductivity on T is calculated as

$$\sigma = \sigma_0 e^{-\frac{E}{RT}} \tag{S1}$$

where σ is the ionic conductivity of the electrolyte/SBE, σ_0 is the ionic conductivity of the electrolyte/SBE at 25 °C, E is the activation energy (J/mol), R is the universal gas constant (J/mol K) and T is the absolute temperature (K).[1]

Table S2: Arrhenius fit parameters of the ionic conductivity data for different electrolyte compositions plotted in Figure 6.

Composition (wt% electrolyte)	Activation Energy (kJ/mol K)	R^2
100%	28.4	1.00
90%	51.8	0.999
80%	47.5	0.999
70%	65.4	0.999
50%	72.3	1.00

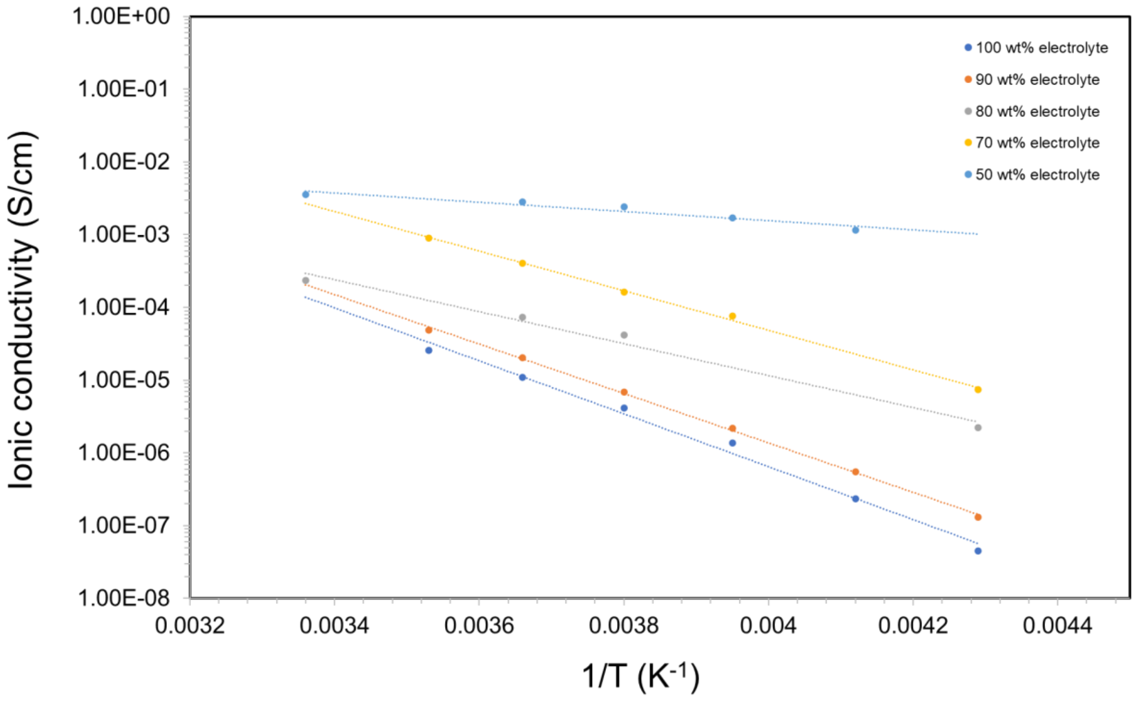

Figure S12: Arrhenius fits over the linear region of the data shown in **Figure 5b**.

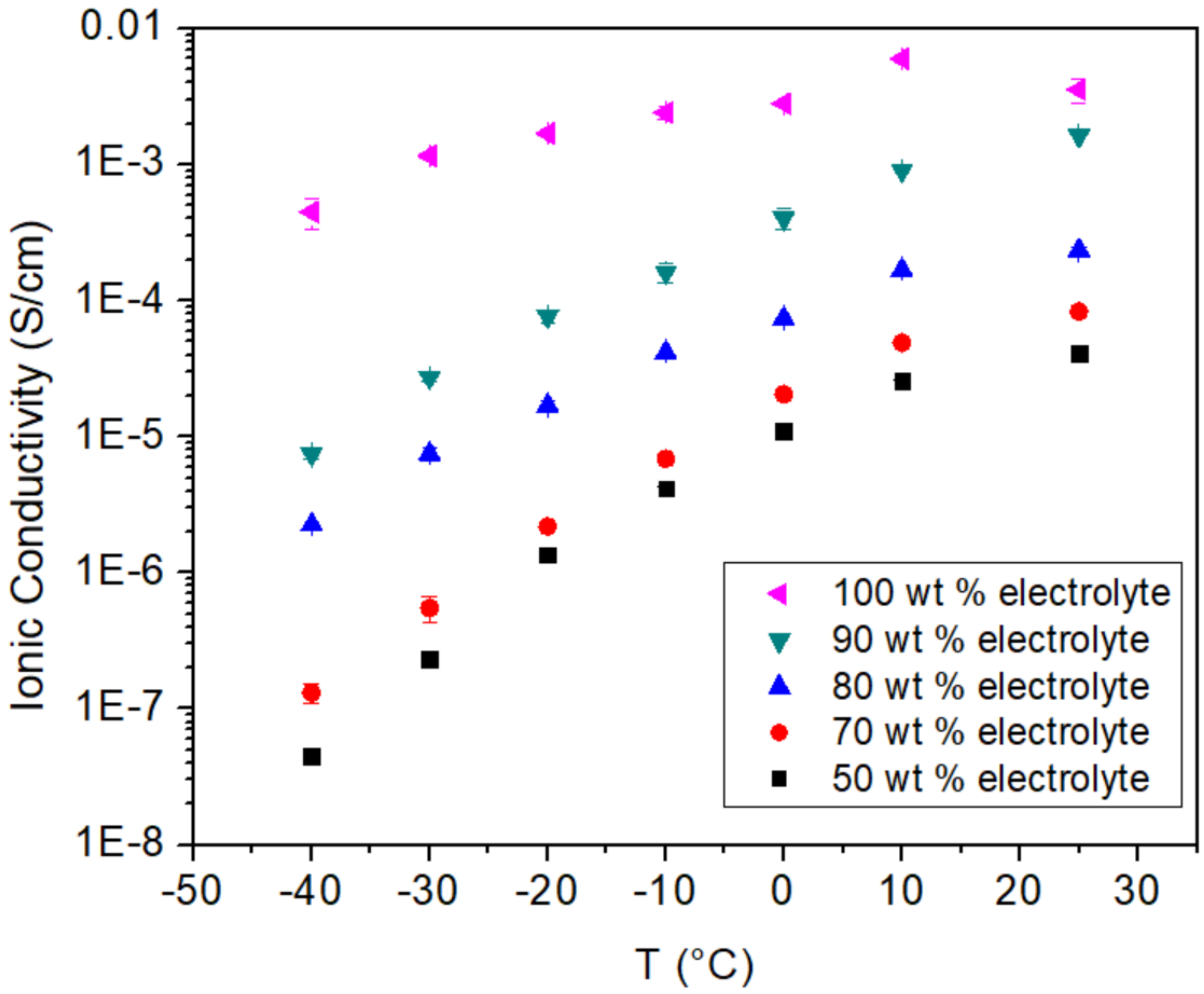

Figure S13: Variation in ionic conductivity with temperature at different electrolyte concentrations. The data corresponds to **Figure 5**.

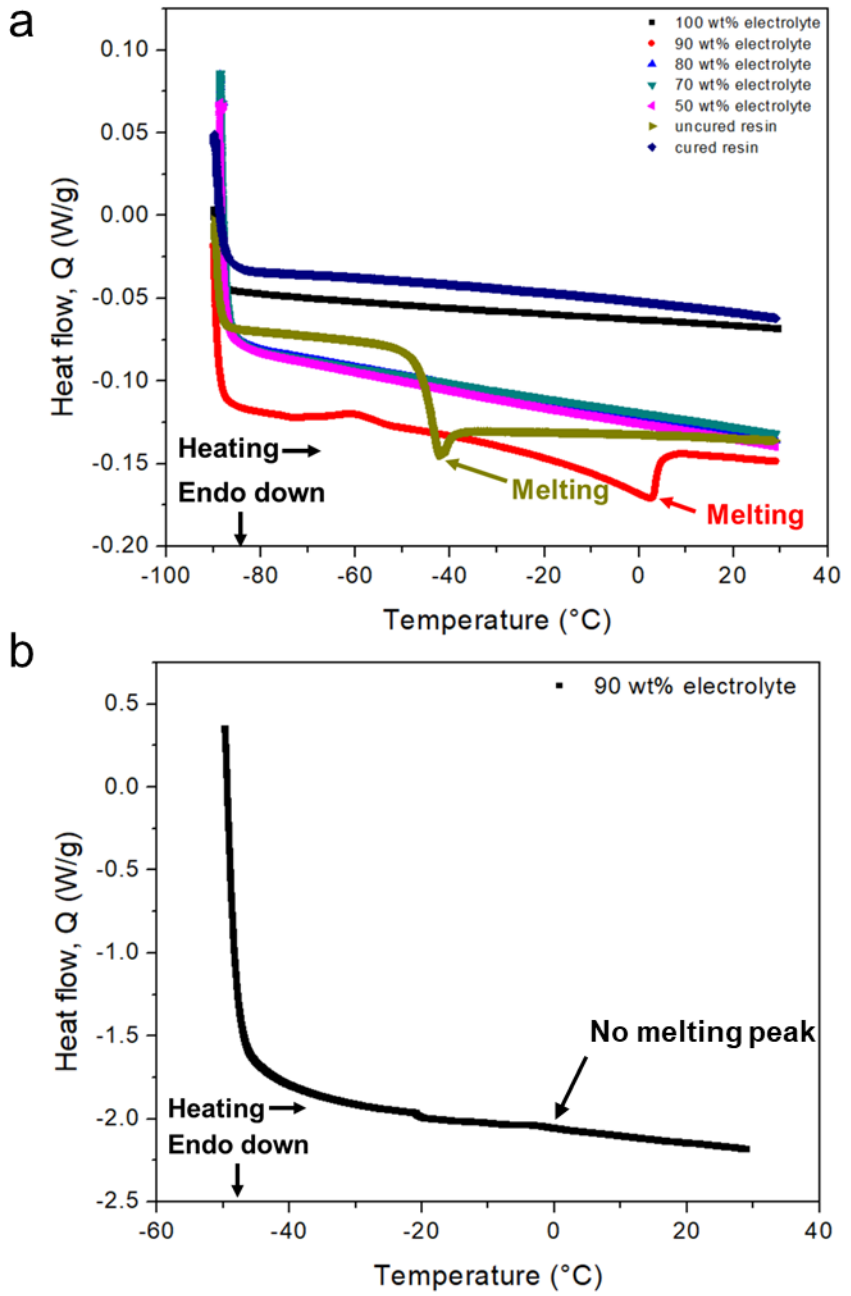

Figure S14: Differential scanning calorimetry (DSC) second heating curves of a) uncured resin, cured resin, and SBE compositions of varying compositions, and b) 90 wt% electrolyte SBE with minimal environmental exposure.

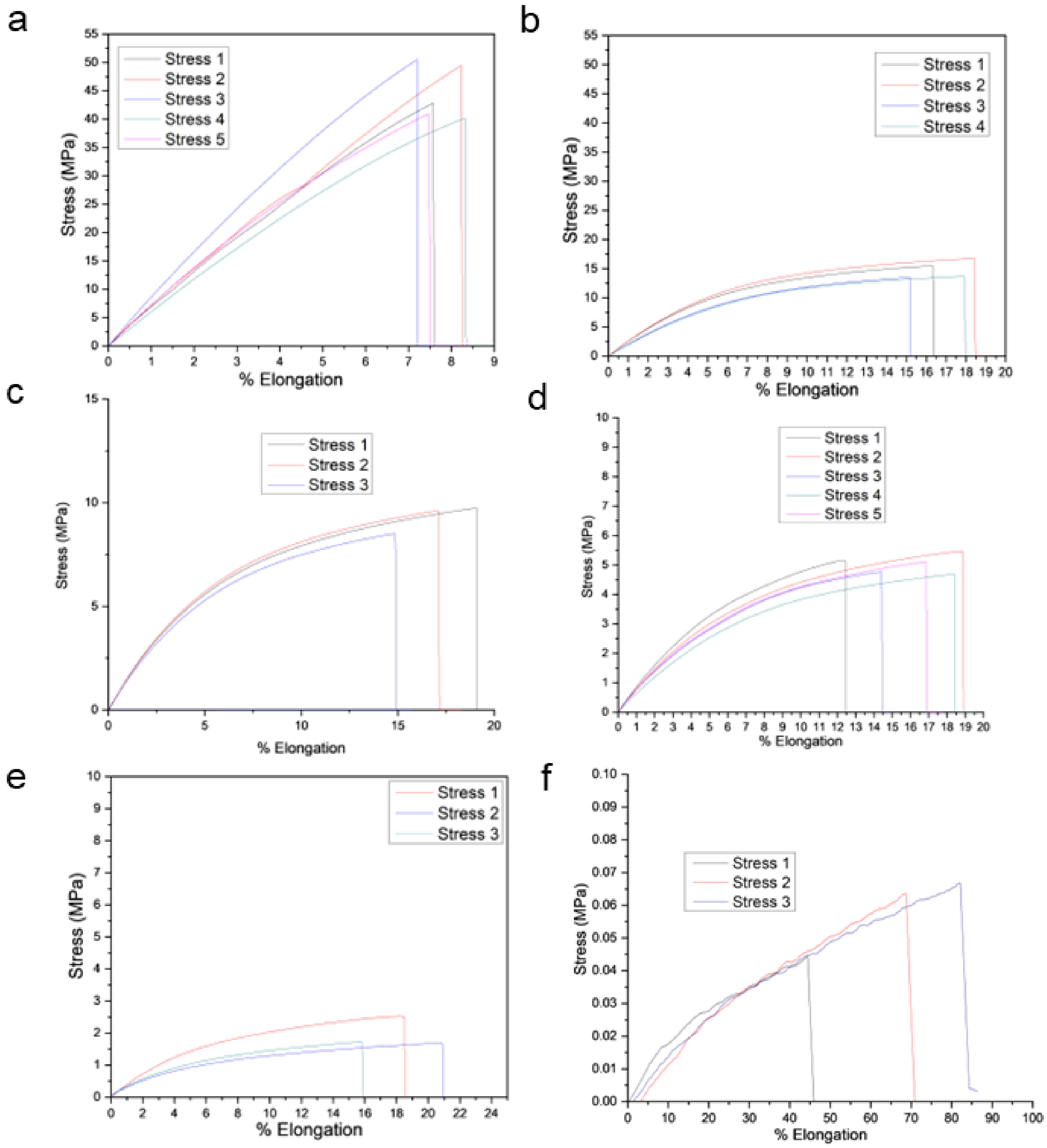

Figure S15: Stress vs. % elongation graphs conducted at 25 °C, a) pure resin, b) 50% electrolyte concentration, c) 60% electrolyte concentration, d) 70% electrolyte concentration, e) 80% electrolyte concentration, f) 90% electrolyte concentration [data smoothed using 1st Order Savitzky-Golay Function].

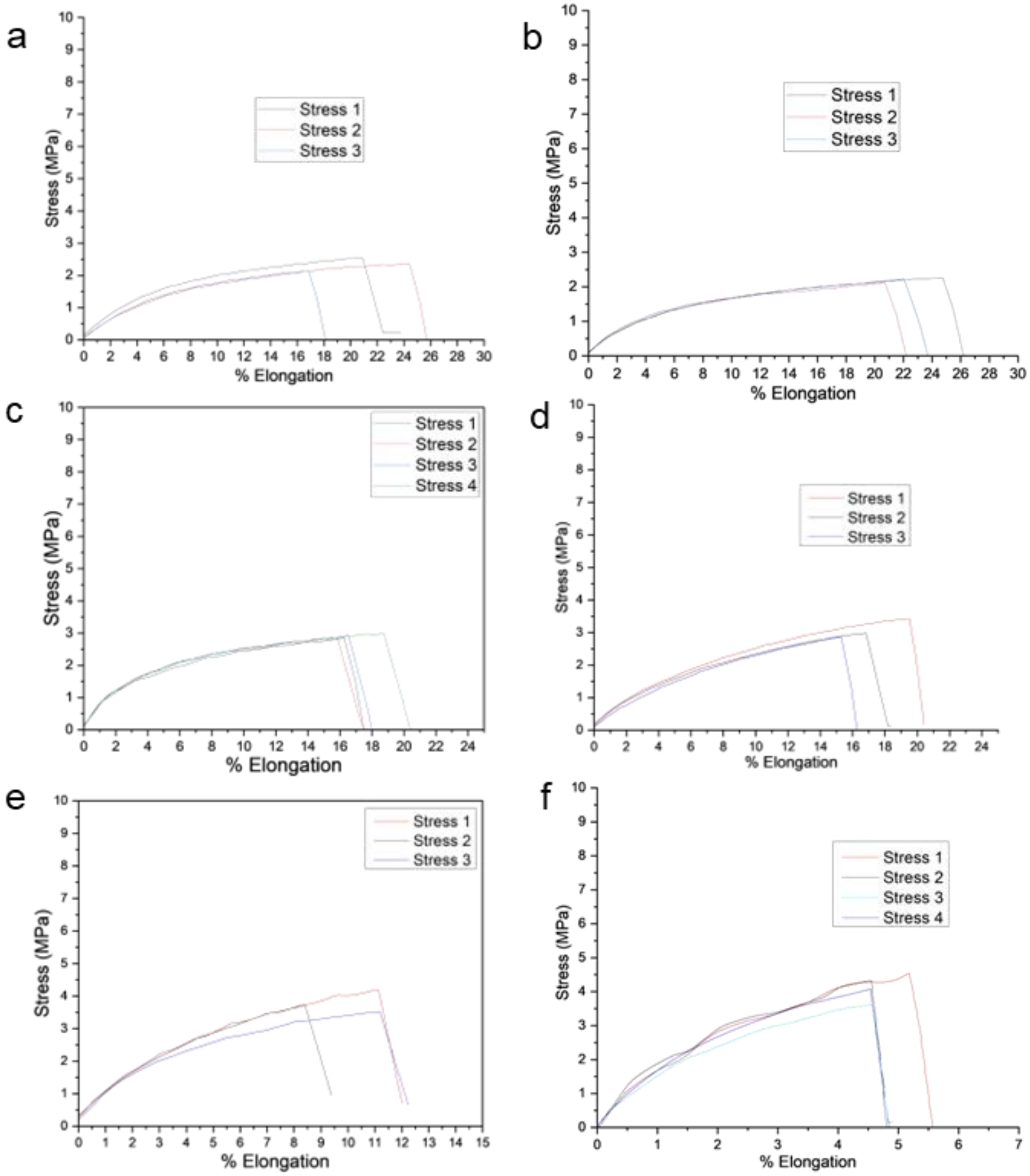

Figure S16: 80% electrolyte concentration stress vs. % elongation smoothed using 1st Order Savitzky-Golay Function, a) at 10 °C, b) at 0 °C, c) at -10 °C, d) at -20 °C, e) at -30 °C, f) at -40 °C.

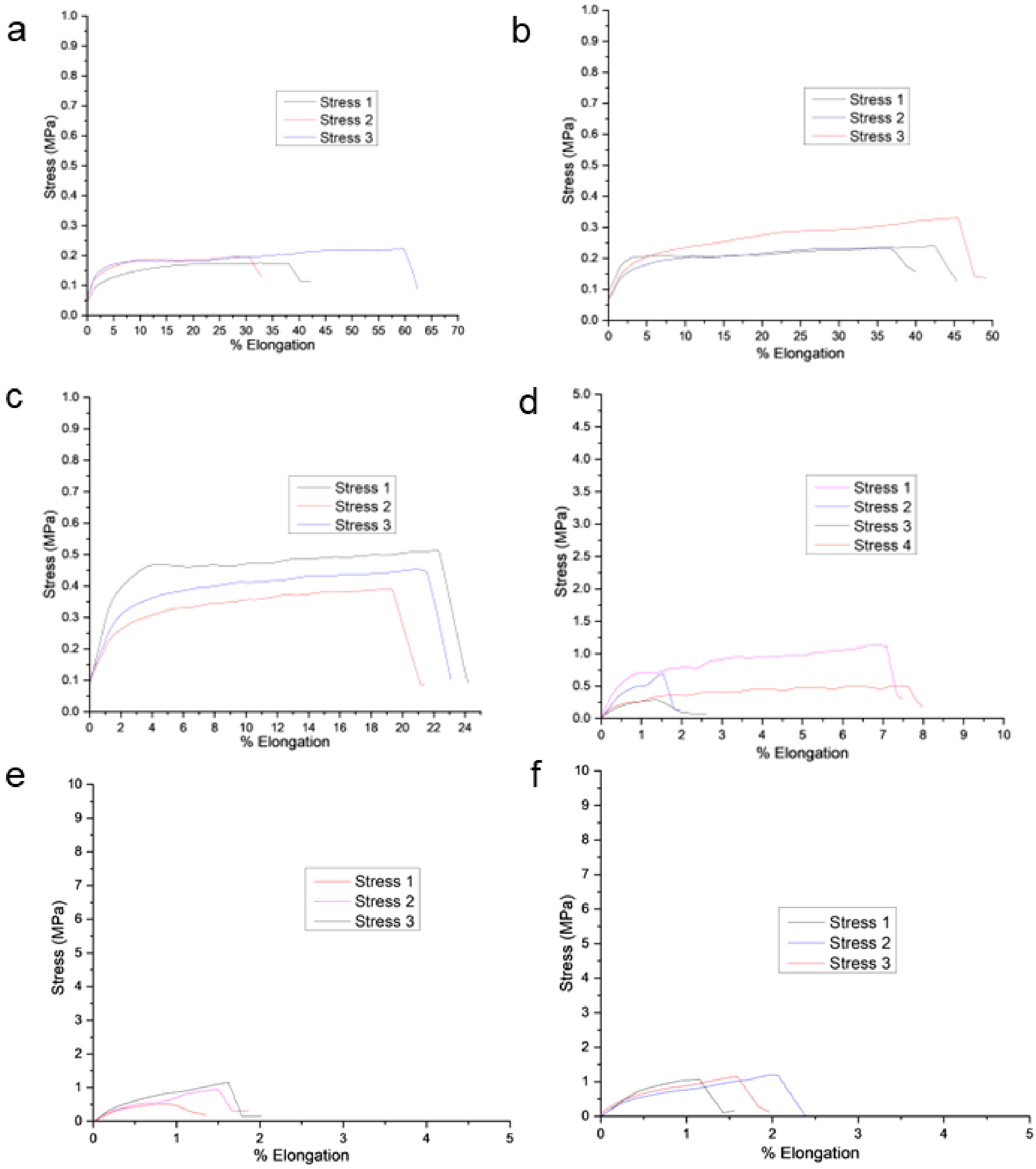

Figure S17: 90% electrolyte concentration stress vs. % elongation smoothed using 1st Order Savitzky-Golay Function, a) at 10 °C, b) at 0 °C, c) at -10 °C, d) at -20 °C, e) at -30 °C, f) at -40 °C.

Table S3: Densities of SBE samples after extraction of liquid electrolyte and density of resin sample. The dimensions of each sample were 1mm x 1mm x 1mm. The density of the SBEs was calculated by dividing its mass after electrolyte extraction by its original volume.

Composition (wt% electrolyte)	Density (g/cm^3)
90%	0.34
80%	0.68
70%	0.84
60%	0.96
50%	1.26
0%	2.59

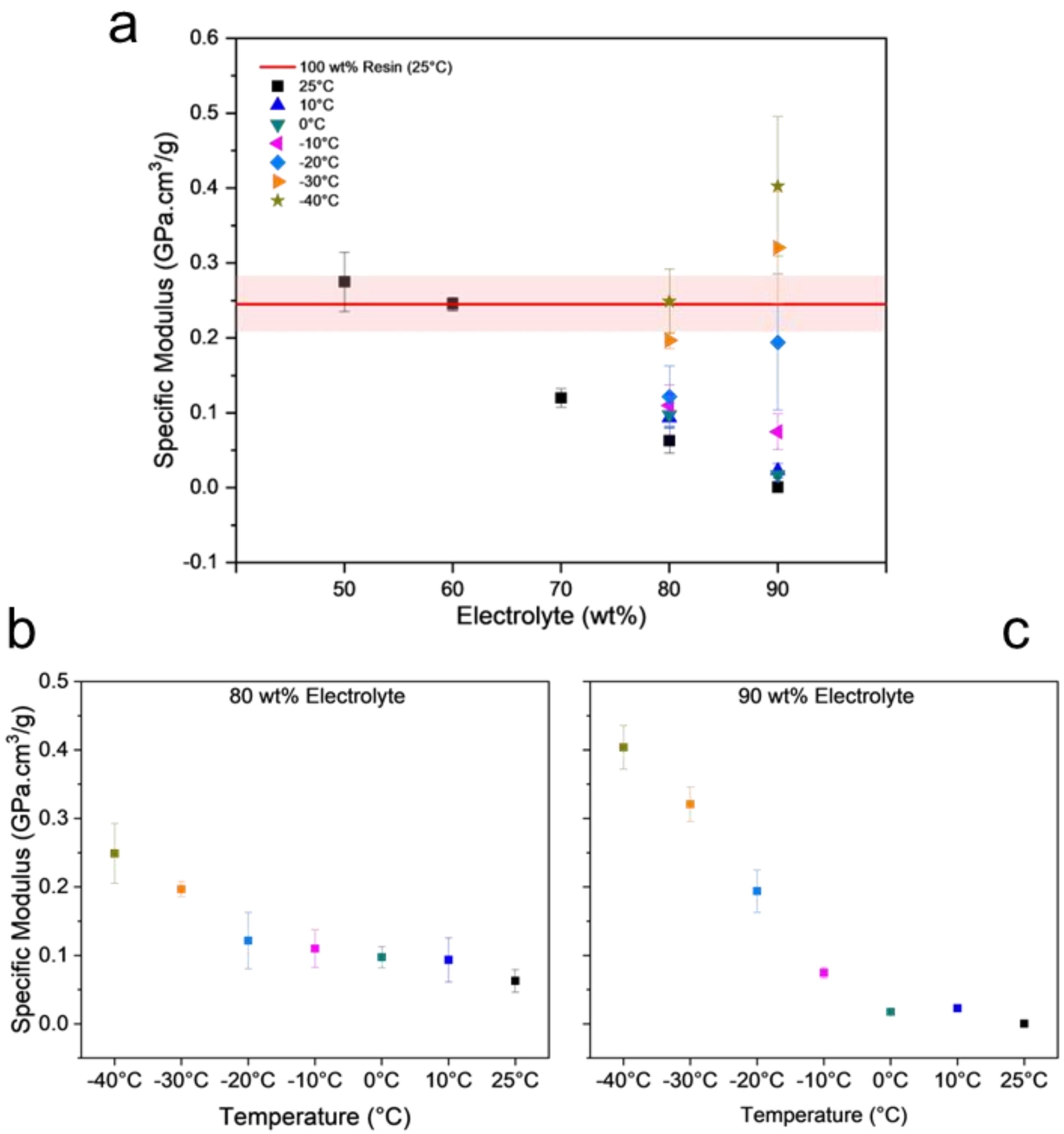

Figure S18: Specific modulus, a) as a function of electrolyte concentration at different temperatures, b) as a function of temperature for 80% electrolyte concentration, c) as a function of temperature for 90% electrolyte concentration. The red strip in (a) represents the pure resin modulus at room temperature (where the center line is the mean value and the shaded region is the associated error). **Note:** The specific modulus predicted by the rule of mixtures is (by definition) the same as the specific modulus of 100 wt% resin.

Table S4: Comparison of ionic conductivity and mechanical strength of different bicontinuous electrolytes studied for both batteries and supercapacitors reported in the literature.[2-10]

Ionic Conductivity (S/cm)	Youngs Modulus (GPa)	Temperature (°C)	Reference
5.50×10^{-5}	0.157	25	[2]
8.60×10^{-5}	0.135	25	[2]
6.74×10^{-4}	1.00	25	[3]
1.48×10^{-5}	0.480	25	[4]
2.30×10^{-4}	0.190	25	[5]
8.00×10^{-4}	0.180	25	[5]
2.00×10^{-4}	0.500	25	[6]
3.50×10^{-4}	0.300	25	[7]
1.00×10^{-4}	0.001	25	[8]
8.00×10^{-5}	Not reported	-30	[9]
3.70×10^{-3}	1.24	25	[10]
2.10×10^{-3}	1.72	25	[10]
1.00×10^{-3}	2.05	25	[10]

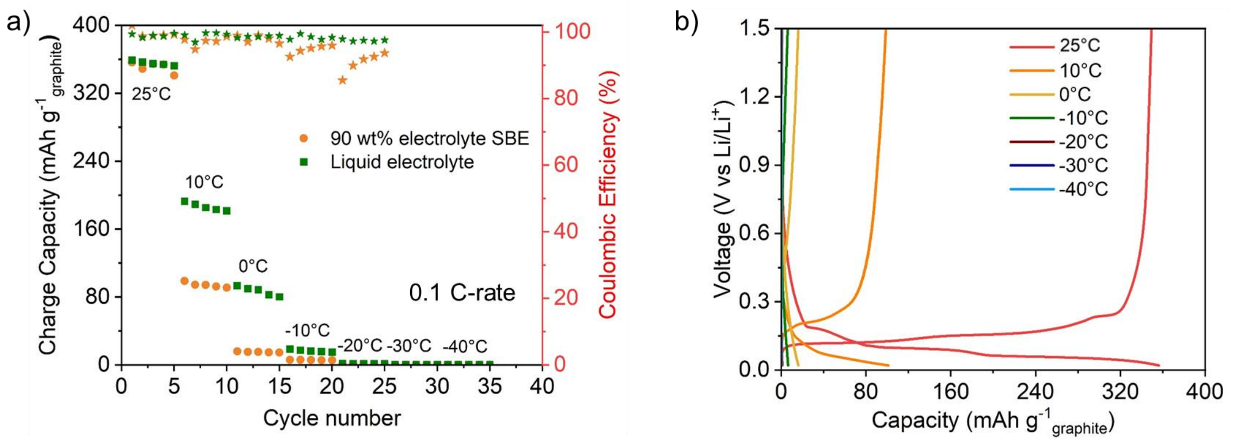

Figure S19: a) GCD cycles for the 100 wt% electrolyte and 90 wt% electrolyte SBE at 25 °C, 10 °C, 0 °C, -10 °C, -20 °C, -30 °C and -40 °C (the stars represent coulombic efficiency while the squares and circles represent charge capacity), b) charge-discharge curves for 90 wt% electrolyte SBE, The current was at 0.1 C. 5 cycles were performed at each temperature. The samples were examined in a coin cell with graphite cathode and lithium metal anode.

REFERENCES

(1) Petrowsky, M.; Frech, R. Temperature Dependence of Ion Transport: The Compensated Arrhenius Equation. *The Journal of Physical Chemistry B* **2009**, *113* (17), 5996-6000. DOI: 10.1021/jp810095g.

(2) Feng, Q.; Yang, J.; Yu, Y.; Tian, F.; Zhang, B.; Feng, M.; Wang, S. The ionic conductivity, mechanical performance and morphology of two-phase structural electrolytes based on polyethylene glycol, epoxy resin and nano-silica. *Materials Science and Engineering: B* **2017**, *219*, 37-44. DOI: https://doi.org/10.1016/j.mseb.2017.03.001.

(3) Zhang, J.; Yan, J.; Zhao, Y.; Zhou, Q.; Ma, Y.; Zi, Y.; Zhou, A.; Lin, S.; Liao, L.; Hu, X.; et al. High-strength and machinable load-bearing integrated electrochemical capacitors based on polymeric solid electrolyte. *Nature Communications* **2023**, *14* (1), 64. DOI: 10.1038/s41467-022-35737-w.

(4) Matsumoto, K.; Endo, T. Confinement of ionic liquid by networked polymers based on multifunctional epoxy resins. *Macromolecules* **2008**, *41* (19), 6981-6986.

(5) Shirshova, N.; Bismarck, A.; Carreyette, S.; Fontana, Q. P. V.; Greenhalgh, E. S.; Jacobsson, P.; Johansson, P.; Marczewski, M. J.; Kalinka, G.; Kucernak, A. R. J.; et al. Structural supercapacitor electrolytes based on bicontinuous ionic liquid–epoxy resin systems. *Journal of Materials Chemistry A* **2013**, *1* (48), 15300-15309, 10.1039/C3TA13163G. DOI: 10.1039/C3TA13163G.

(6) Kwon, S. J.; Kim, T.; Jung, B. M.; Lee, S. B.; Choi, U. H. Multifunctional Epoxy-Based Solid Polymer Electrolytes for Solid-State Supercapacitors. *ACS Applied Materials & Interfaces* **2018**, *10* (41), 35108-35117. DOI: 10.1021/acsami.8b11016.

(7) Chopade, S. A.; Au, J. G.; Li, Z.; Schmidt, P. W.; Hillmyer, M. A.; Lodge, T. P. Robust Polymer Electrolyte Membranes with High Ambient-Temperature Lithium-Ion Conductivity via Polymerization-Induced Microphase Separation. *ACS Applied Materials & Interfaces* **2017**, *9* (17), 14561-14565. DOI: 10.1021/acsami.7b02514.

(8) Westover, A. S.; Baer, B.; Bello, B. H.; Sun, H.; Oakes, L.; Bellan, L. M.; Pint, C. L. Multifunctional high strength and high energy epoxy composite structural supercapacitors with wet-dry operational stability. *Journal of Materials Chemistry A* **2015**, *3* (40), 20097-20102, 10.1039/C5TA05922D. DOI: 10.1039/C5TA05922D.

(9) Shirshova, N.; Bismarck, A.; Greenhalgh, E. S.; Johansson, P.; Kalinka, G.; Marczewski, M. J.; Shaffer, M. S. P.; Wienrich, M. Composition as a Means To Control Morphology and Properties of Epoxy Based Dual-Phase Structural Electrolytes. *The Journal of Physical Chemistry C* **2014**, *118* (49), 28377-28387. DOI: 10.1021/jp507952b.

(10) Qi, G.; Cui, Q.; Zhang, B.; Du, S. A carbon fiber lamina electrode based on macroporous epoxy with vertical ion channels for structural battery composites. *Composite Structures* **2023**, *304*, 116425. DOI: https://doi.org/10.1016/j.compstruct.2022.116425.
